# Composite tumor of metanephric adenoma and Wilms’ tumor of the kidney: A case report and review of the literature

**DOI:** 10.3892/ol.2013.1148

**Published:** 2013-01-22

**Authors:** PENGCHENG ZHU, FEI YAN, ZIXUAN YANG, LI MENG, QILIN AO

**Affiliations:** 1 Institute of Pathology, Tongji Hospital, Tongji Medical College, Huazhong University of Science and Technology, Wuhan 430030;; 2 Department of Oncology, Zhongshan Hospital of Hubei Province, Wuhan 430000, P.R. China

**Keywords:** composite tumor, metanephric adenoma, Wilms’ tumor, differential diagnosis

## Abstract

Metanephric adenoma (MA) and Wilms’ tumor (WT) are two distinct types of renal tumors. Composite MA and WT of the kidney are extremely rare. Here, a rare case of composite MA and WT of the kidney in a 36-year-old male is described. MA and WT each have their own histopathological features, respectively, and they focally share morphological similarities, which can be a diagnostic challenge. Immunohistochemistry is useful in the differential diagnosis of MA and WT. The histopathological features and differential diagnosis of the composite tumor are emphasized here to promote a better and broader understanding of this less understood subject.

## Introduction

Metanephric adenoma (MA) is a rare benign renal tumor which is related to the developing proximal tubule of the fetal kidney or nephrogenic rests. The tumor generally occurs in female adults and occasionally in children (1). Wilms’ tumor (WT), also known as nephroblastoma, is the most common malignant tumor which originates from developing nephrogenic tissue, occurring in the genitourinary tract in children (2). The nature of metanephric adenoma is still not entirely clear, although some investigators believed that it is related to WT because these two tumors sometimes share morphologic similarities and immunoreactivity for Wilms’ tumor gene1 (WT-1) (3,4).

Here, a rare composite tumor with MA and WT histopathologic features of the kidney in a male adult is reported. This case represents the third report of this composite tumor of MA and WT and may present clues to elucidate the pathogenesis of MA and WT (5,6). The study was approved by the Ethics Committee of Tongji hospital, Tongji Medical College, Huazhong University of Science and Technology, Wuhan, China. Written informed consent was obtained from the patient.

## Case report

A 36-year-old male visited the Department of Urology, Tongji Hospital, Wuhan, China, for evaluation of his renal area following approximately 4 months of illness. The medical history and review of symptoms were noncontributory.

**Figure 1:**
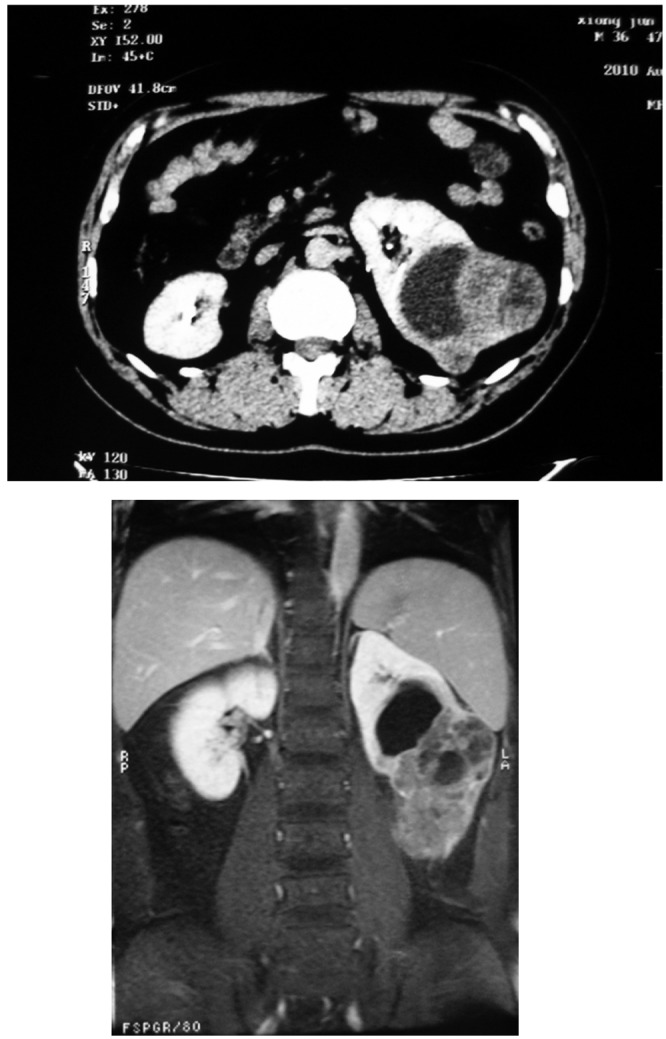
Computed tomography and magnetic resonance imaging showing a large solid mass located in the middle and lower pole of the left kidney with hydronephrectasis.

Computed tomography (CT) and magnetic resonance (MR) scan revealed an expansile, solid mass measuring ∼10 cm in the middle lower pole of the left kidney (Fig. 1). The mass was relatively ill-marginated. The patient was admitted for surgical intervention and the lesion was excised under general anesthesia.

### Pathological examination

Specimens from the mass were fixed in 10% formalin, embedded in paraffin, sectioned, and stained with hematoxylin and eosin (H&E) by means of routine procedures. Immunostaining was performed on 4-*μ*m-thick sections using the standard avidin-biotin complex technique. A panel of antibodies (Table I) was used.

Immunostaining was performed by an enhancement method based on repetitive microwave heating of slides that were placed into 0.01 M citrate buffer at pH 6.0. Binding of primary antibodies was visualized with an Envision two-step method. Diaminobenzidine was used as the chromogen. Nuclei were stained with Mayer’s hematoxylin. Appropriate positive and negative controls were included.

### Results

**Figure 2:**
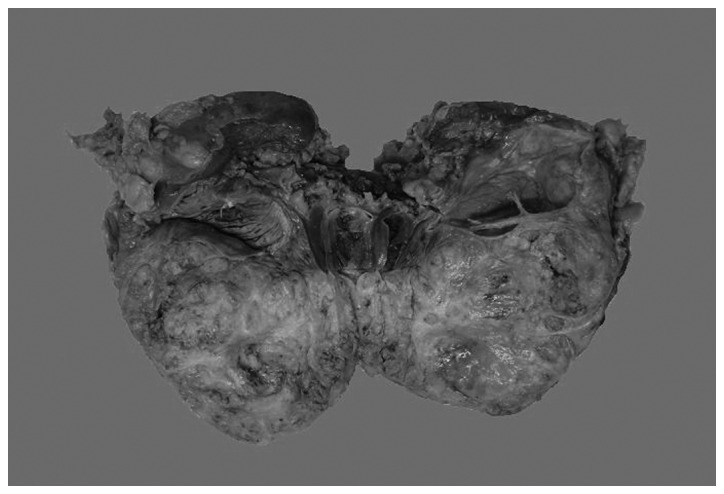
The cut surfaces of the tumor were yellowish gray and solid with an ill-circumscribed border.

Macroscopically, the specimen was ill-circumscribed and measured 10 cm at its greatest dimension, and hydronephrectasia could be seen in the left kidney due to the occupation of the tumor; the cut surfaces were yellowish gray and solid (Fig. 2). There was no obvious necrosis or hemorrhage. The adjacent renal parenchyma appeared normal. The renal capsule was intact.

**Figure 3:**
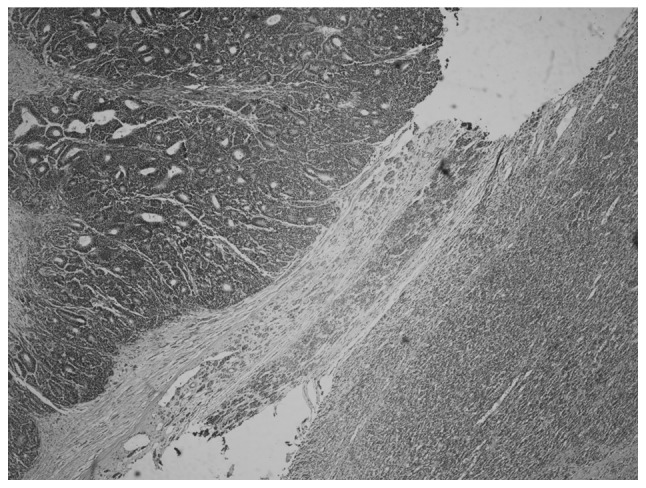
The composite tumor showing metanephric adenoma (MA; right side) along with Wilms’ tumor (WT; left side) separated by tiny fibrous stroma (hematoxylin and eosin; magnification, ×100).

**Figure 4:**
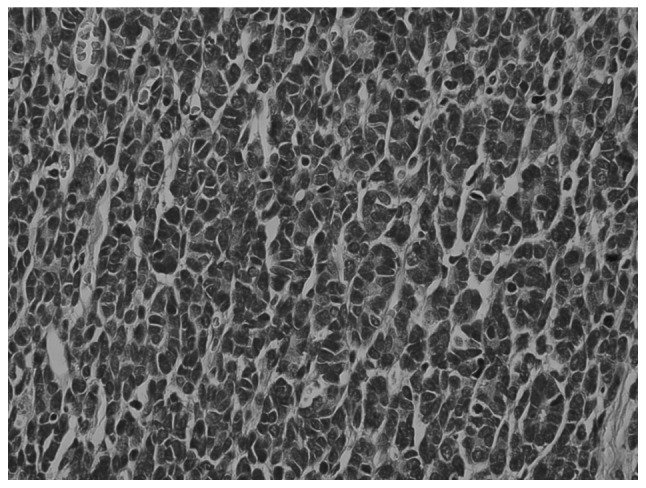
The metanephric adenoma (MA) area showed ordered array of small, tightly packed acini and tubules. Tumor cells are small and uniform with round to oval nuclei and scant cytoplasm (hematoxylin and eosin; magnification, ×400).

Microscopically, the tumor consisted of two distinct areas of MA and WT that were separated by a band of fibrous stroma (Fig. 3). MA showed a pushing border with no capsule and an ordered array of small, tightly packed acini and tubules separated by acellular stroma (Fig. 4), but no papillary structure. The tumor cells were small and uniform with round to oval nuclei and scant cytoplasm. Nuclei showed delicate chromatin and inconspicuous nucleoli; mitotic activity was rare to absent.

**Figure 5:**
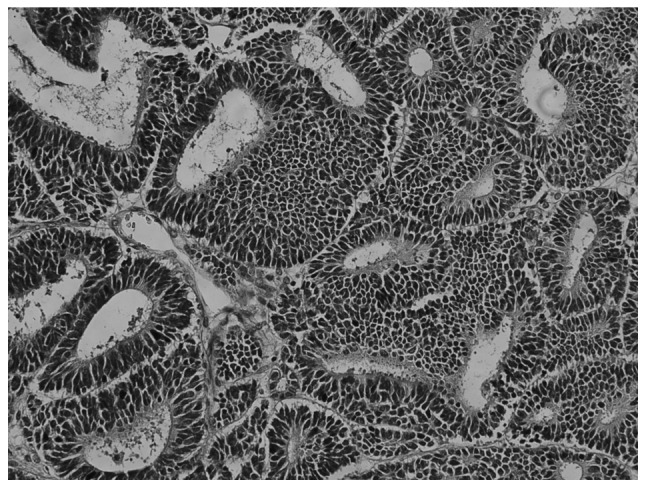
Wilms’ tumor (WT) area showed predominantly epithelial components which are in the form of poorly formed tubules to well-developed tubular structures. The typical blastemal and stromal components were rare (hematoxylin and eosin; magnification, ×400).

The WT area showed a predominantly epithelial component which was in the form of either poorly formed or well-developed tubules (Fig. 5) The typical blastemal and stromal components were rarely observed.

**Figure 6:**
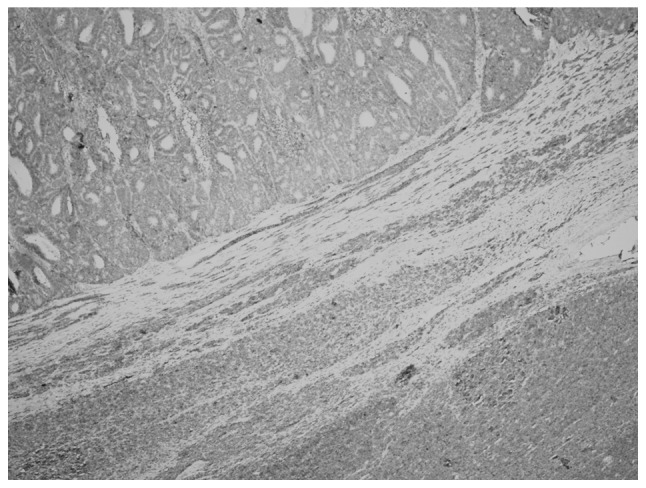
Tumor cells showing CD57 expression in the metanephric adenoma area (right side) as compared with negativity in the Wilms’ tumor area (left side) (immunohistochemistry; magnification, ×100).

**Figure 7:**
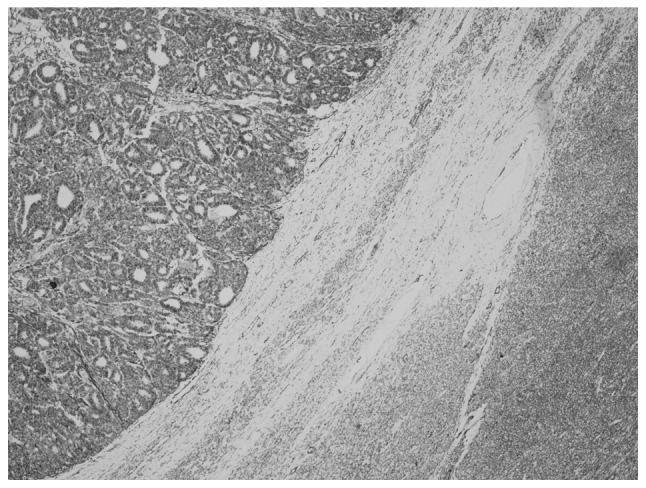
Tumor cells showing WT-1 expression in the Wilms’ tumor area (left side) as compared with negativity in the metanephric adenoma area (right side) (immunohistochemistry, magnification, ×100).

**Figure 8:**
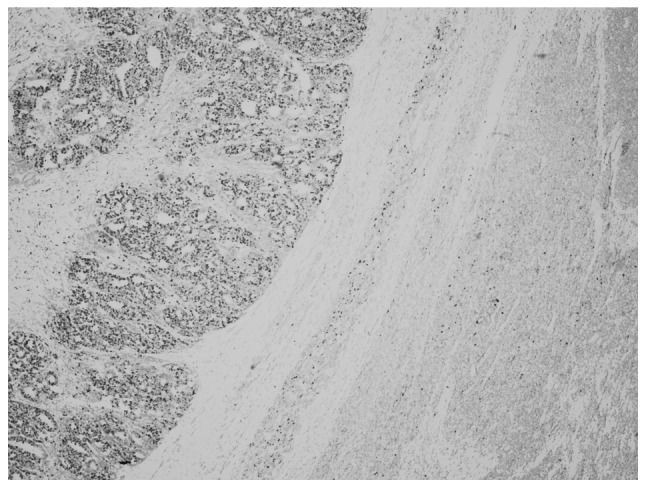
The Ki67 labeling index was significantly higher in the Wilms’ tumor area (left side) as compared with that of the metanephric adenoma area (right side) (immunohistochemistry; magnification, ×100).

In the MA area, tumor cells showed positive diffuse staining for CD57 (Fig. 6), WT-1 (Fig. 7), focal staining for vimentin, pan CK, CK7 and CK8/18, but negative staining for epithelial membrane antigen (EMA). The Ki67 labeling index (Fig. 8) was 2%. In the WT area, tumor cells stained diffusely for WT-1 (Fig. 7), CK8/18, focally for vimentin, pan CK, EMA, CD57 (Fig. 6), but were negative for CK7. The Ki67 labeling index (Fig. 8) was 50–60%.

## Discussion

Composite renal tumors are rarely reported. The most commonly described association is of Wilms’ tumor and renal cell carcinoma (7). Composite tumors of MA and WT of the kidney are extremely rare (5, 6).

MA is a well-described rare benign renal tumor, predominantly occurring in adult females, and seldom observed in children. WT is the most common malignant renal tumor in children but it is rare in adults. Histologically, MA is composed of tightly packed uniform small epithelial cells in acinar, solid and tubular configurations with small regular nuclei, a high nucleus to cytoplasm ratio, but low mitotic figures.

Wilms’ tumors are typically composed of a mixture of primitive blastemal cells, epithelial cells and mesenchymal elements. In this case, the epithelial component in the WT area was predominant; it was therefore named epithelial-predominant WT (8). Due to overlapping morphological features, histopathological examination of MA often prompts an initial diagnosis of epithelial-predominant WT.

Since MA and epithelial-predominant WT could share microscopical similarities, immunohistochemistry (IHC) played a significant contributory role in the distinction of these two entities. Although CD57 is not particular for diagnosis of MA, it is helpful in the differential diagnosis between MA and epithelial-predominant WT (9, 10). In MA, the epithelial cells are positive for CD57, while the tumor cells in epithelial-predominant WT are negative. The Ki67 labeling index is significantly lower in MA compared with epithelial-predominant WT, which also supports these two areas belonging to distinct lesions (6). The two distinct areas had individual histopathological features and special IHC staining, both of which contributed to the reaffirmation of the histomorphological diagnosis of composite MA and epithelial-predominant WT.

Both MA and epithelial-predominant WT were positive for WT-1, leading to the theory that the two could be linked (9), and MA could even be a more hyperdifferentiated, mature form of WT (11). Since there was no other supporting evidence it was hypothesized that they were two distinct entities.

Another diagnostic challenge is differentiating MA from the solid variant form of papillary renal cell carcinoma (PRCC) based on histologic features alone. IHC is helpful since PRCC is negative for CD57 and WT-1 (12,13).

To date, the genetic basis of MA remains largely unknown since previous reports have given conflicting results (14–16). BRAF mutation has been reported in 90% cases in a series of MA studies but it is rarely detected in other kidney tumors, including WT. Testing for the BRAF mutation could, therefore, serve as a potential diagnostic tool for MA (17).

In summary, a rare case of composite MA and epithelial-predominant WT in an adult kidney is presented. Although there were overlapping morphological features, it was possible to differentiate MA from WT based on the morphologic features and IHC staining. This case also offered additional support to the hypothesis that these two tumors are related.

## Figures and Tables

**Table I:** Antibodies and dilutions used in the evaluation of composite metanephric adenoma and Wilms’ tumor of the kidney.

Antibody	Dilution	Source	Antigen retrieval
Vimentin	1:20	Dako	Heat
AE1/AE3	1:20	Dako	Heat
CD57	1:50	Dako	Heat
WT-1	1:200	Dako	Heat
EMA	1:100	Dako	Heat
CK7	1:200	Dako	Heat
CK8/18	1:100	Dako	Heat
Ki67	1:40	Dako	Heat
